# Lower circulating levels of CTRP12 and CTRP13 in polycystic ovarian syndrome: Irrespective of obesity

**DOI:** 10.1371/journal.pone.0208059

**Published:** 2018-12-12

**Authors:** Mehrnoosh Shanaki, Nariman Moradi, Reza Fadaei, Zahra Zandieh, Parisa Shabani, Akram Vatannejad

**Affiliations:** 1 Department of Medical Laboratory Sciences, School of Allied Medical Science, Shahid Beheshti University of Medical Sciences, Tehran, Iran; 2 Department of Biochemistry, Faculty of Medicine, Iran University of Medical Sciences, Tehran, Iran; 3 Sleep Disorders Research Center, Kermanshah University of Medical Sciences, Kermanshah, Iran; 4 Cellular and Molecular Research Center, School of Medicine, Iran University of Medical Sciences, Tehran, Iran; 5 Shahid Akbar Abadi Clinical Research Development Unit (ShACRDU), Iran University of Medical Sciences (IUMS), Tehran, Iran; 6 Department of Biochemistry, Faculty of Medicine, Semnan University of Medical Sciences, Semnan, Iran; 7 Department of Cellular and Molecular Nutrition, School of Nutritional Sciences and Dietetics, Tehran University of Medical Sciences, Tehran, Iran; 8 Students' Scientific Research Center (SSRC), Tehran University of Medical Sciences, Tehran, Iran; John Hopkins University School of Medicine, UNITED STATES

## Abstract

Altered production of adipokines is suggested to play a pivotal role in the pathogenesis of polycystic ovarian syndrome (PCOS). C1q/TNF-related proteins (CTRPs) play diverse roles in regulation of metabolism in physiologic and pathologic conditions. In the present study, we assessed serum concentrations of adiponectin, CTRP12, and CTRP13 in individuals with PCOS and those without PCOS. We also evaluated the possible association of these adipokines with metabolic and hormonal variables. A total of 171 premenopausal women (86 with PCOS and 85 without PCOS) enrolled in this study. Serum levels of adiponectin, CTRP12, and CTRP13 were measured. The results showed significantly lower serum concentrations of adiponectin, CTRP12, and CTRP13 in PCOS women compared to non-PCOS women. This difference remained significant after controlling for age, body mass index (BMI), and Homeostasis Model Assessment of Insulin Resistance (HOMA-IR). However, we did not observe any significant differences in serum levels of adiponectin, CTRP12, and CTRP13 between the overweight/obese and normal weight subgroups in PCOS and non-PCOS women. Multiple linear regression analysis showed associations of CTRP12 with adiponectin and BMI with CTRP13 in both the PCOS and non-PCOS groups. CTRP12 was significantly associated with BMI and adiponectin in the non-PCOS group, and fasting blood glucose (FBG) and CTRP13 in the PCOS group. Our results indicated that decreased adiponectin, CTRP12, and CTRP13 levels, regardless of obesity, could independently predict PCOS. This finding suggested a novel link between adipokines and PCOS.

## Introduction

Polycystic ovarian syndrome (PCOS) is one of the most common endocrine disorders, affecting 4%–21% of reproductive age women [1]. This syndrome is characterized by a constellation of signs and symptoms that include hyperandrogenism, oligo-ovulation or anovulation, and polycystic ovaries on ultrasound [1, 2].

The pathophysiology of PCOS also extends to metabolic abnormalities, as most PCOS individuals are obese [3]. PCOS is also associated with glucose intolerance, which is considered to be the main cause of the clinical characteristics of PCOS [4, 5]. Obese PCOS patients are more likely to experience infertility [6]. Obesity contributes to infertility in PCOS patients through multiple mechanisms such as amplification of hyperandrogenism, alteration of luteinizing hormone (LH) secretion, and higher insulin resistance [6]. Obesity is accompanied by an alteration in the secretory profile of adipose tissue, which influences the whole-body metabolic state [7]. Disturbed adipokine secretion plays an important role in the pathogenesis of PCOS through multiple mechanisms, such as modulation of the activity of the pituitary–ovarian axis [8, 9].

Previous studies have reported dysregulation of adipokines in PCOS patients [10–13]. Among the adipokines, adiponectin is a protein produced predominantly by adipocytes. Adiponectin improves insulin sensitivity, and exerts anti-atherogenic and anti-inflammatory effects [14]. Several studies have indicated lower serum levels of adiponectin in PCOS patients [15–17]. Numerous proposed beneficial effects of adiponectin on the reproductive system include its influence on the female reproductive endocrine axis through adiponectin specific receptors expressed in the hypothalamus and hypophysis [18]. Additionally, adiponectin has been shown to modulate ovulation and play a role in regulation of steroid hormone synthesis [18, 19]. Adiponectin belongs to the C1q/TNF-related protein (CTRPs) family, a recently identified family of adipokines [20]. CTRPs are expressed by adipose and other tissues; most circulate in the plasma and play diverse roles in regulation of metabolism under physiologic and pathologic conditions [20, 21]. CTRPs share similar globular domains and metabolic functions as adiponectin [20]. In this regard, previous studies suggested the possible compensatory effects of CTRPs in the absence of adiponectin [20, 22].

While several studies have indicated the direct role of adiponectin in the pathogenesis of PCOS, there is little studies that addressed the association of CTRPs with PCOS. In this regard, a recent study has demonstrated that serum levels of CTRP9 was not significantly different between PCOS patients and healthy individuals [23]. Furthermore, previous studies have reported lower circulating levels of CTRP3 and CTRP12 in PCOS patients [24, 25]. It seems that dysregulation of CTRP family members may contribute to the pathogenesis of PCOS. Our previous studies have indicated lower serum levels of CTRP13 in other metabolic disorders including fatty liver, diabetes and coronary artery disease [26–28].In humans, CTRP13 is expressed predominantly by adipose tissue [22]. Obese mice showed dysregulated CTRP13 expression that was sex-specific. While CTRP13 expression increased significantly in obese male mice, its expression remained unchanged in obese female mice [22]. Rosiglitazone, an insulin sensitizing drug, increased expression of CTRP13 in 3T3-L1 adipocytes [22]. The increased expression of CTRP13 in obese male mice was attributed to a compensatory defense against further obesity-derived insulin resistance [22]. But, CTRP13 is also expressed by brain and it has been demonstrated that CTRP13 may play a role in forming hypothalamic feedback which can regulate food intake [29]. Other clinical studies reported lower serum concentrations of CTRP12 in metabolic-related disorders [24, 30]. In humans, CTRP12 is expressed mainly by adipose tissue [31]. CTRP12 expression in adipose tissue correlates well with CTRP12 serum concentrations [31, 32]. Previous studies have indicated disturbed expression of CTRP12 in the obesity state and its upregulation by insulin, rosiglitazone and metformin [24, 31, 33]. Recombinant CTRP12 decreased blood glucose levels and improve insulin sensitivity in obese and diabetic mice [31].

CTRP12 and CTRP13 play important roles in metabolic homeostasis [22, 31]. Hence, it has been of particular interest to investigate their possible association with metabolic disorders in clinical studies. There are a paucity of studies that assessed the associations of CTRP12 and CTRP13 with PCOS. In the current study, we aimed to determine the serum concentrations of adiponectin, CTRP12, and CTRP13 in PCOS and non-PCOS individuals. We also evaluated the possible association of these adipokines with hormone, and metabolic parameters.

## Materials and methods

We recruited the study participants from individuals who presented to Treata Hospital, Iran from May 2017 to January 2018. The Shahid Beheshti University of Medical Sciences Ethics Committee approved this study. In total, 171 premenopausal women (86 with PCOS and 85 without PCOS) enrolled in this study. The women were between 20 and 40 years of age and had body mass index (BMI) values between 17 and 35 kg/m^2^. All participants gave written informed consent before entering the study. PCOS patients were diagnosed according to the Rotterdam criteria. The Rotterdam criteria confirms a diagnosis of PCOS when at least two of the following three criteria are met: Oligo-ovulation or anovulation, hyperandrogenism, and the presence of polycystic ovaries on ultrasound [34]. Non-PCOS women had regular menstrual cycles, absence of hirsutism and other manifestations of hyperandrogenism, and no history of infertility. Women with diabetes mellitus, cardiovascular diseases, hypertension, current pregnancy, and history of endocrine disorders were excluded from the study. None of the participants were on hormones or antiandrogen therapies, or any medication known to influence glucose metabolism or endocrine parameters.

Anthropometric parameters of the study subjects that included age, height, and weight were measured. BMI was calculated as body weight (kg) divided by the square of the individual’s height (m^2^). Study participants provided venous blood samples after an overnight fast. Fasting blood glucose (FBG) and lipid profiles that included triglycerides (TG), total cholesterol (TC), low density lipoprotein cholesterol (LDL-C), and high density lipoprotein cholesterol (HDL-C) were measured by automated enzymatic methods and commercial kits (Pars Azmoon, Iran). Fasting serum insulin was measured by an ELISA kit (Monobind, Inc., USA; catalog number: 5825–300). Insulin resistance was determined using the Homeostasis Model Assessment of Insulin Resistance (HOMA-IR), calculated as [FBG (mg/dL)] × [fasting serum insulin (μU/mL)]/405 [35]. We used ELISA kits to assess follicle-stimulating hormone (FSH; Pishtaz Teb, Iran; catalog number: PT-FSH-96), LH (Pishtaz Teb, Iran; catalog number: PT-LH-96), and free testosterone (FT; Monobind, Inc., USA; catalog number: 5325–300) levels.

Serum adiponectin levels were assessed using a human adiponectin ELISA kit (Aviscera Bioscience, Inc., USA; catalog number: SK00010-01) according to the methods specified by the manufacturer. Sensitivity for adiponectin was 31 pg/ml, with an intra-assay coefficient of variation of 4%-8% and inter-assay coefficient of variation of 8%-12%. Serum levels of CTRP12 and CTRP13 were also measured using human CTRP12 (Aviscera Bioscience, Inc.; catalog number: SK00392-06) and CTRP13 ELISA kits (Aviscera Bioscience, Inc.; catalog number: SK00333-06). CTRP12 sensitivity was 10 pg/ml and the sensitivity for CTRP13 was 2 ng/ml. The intra-assay coefficient of variation for CTRP12 was 6%-8% and the inter-assay coefficient of variation was 8%-12%. CTRP13 had an intra-assay coefficient of variation of 4%-6% and an inter-assay coefficient of variation of 8%-12%.

All analyses were performed using SPSS 16 (SPSS, Chicago, IL, USA). The Kolmogorov-Smirnov test was conducted to identify normal distribution of the variables. Continuous variables are presented as mean ± standard deviation (SD) or median and interquartile range as appropriate. The student's t-test and Mann-Whitney U test were used to determine differences between PCOS and non-PCOS groups. After subdividing the two groups into overweight/obese and normal weight, we performed ANOVA and Kruskal-Wallis with Bonferroni corrections post hoc to determine the difference among the subgroups. Then, ANCOVA was performed to remove the effects of potential confounders. Non-normally distributed variables were log-transformed to approximate normality before further analyses. Correlations between continuous variables were determined using Pearson’s correlations. Subsequently, when individual correlations achieved statistical significance, we entered the variables into multiple linear regression analysis with adiponectin, CTRP12, and CTRP13 as dependent variables. For all data, a p-value of <0.05 was considered statistically significant.

## Results

Table 1 lists the clinical and laboratory characteristics of the PCOS and non-PCOS women, including metabolic and hormone profiles. Age and BMI were comparable between PCOS and non-PCOS women. Women with PCOS had higher serum insulin levels and HOMA-IR, which reflected higher insulin resistance (p<0.001). PCOS women also had higher serum levels of LH (p<0.001), FSH (p = 0.044), FT, and LH/FSH ratio (p<0.001) compared to non-PCOS women. FBG, HDL-C, LDL-C, TC, and TG did not significantly differ between PCOS and non-PCOS women.

**Table 1:** Baseline clinical characteristics of non-PCOS and PCOS subjects.

Variables	Non-PCOS (n = 85)	PCOS (n = 86)	p-value
Age (years)	29.79 ± 4.30	29.93 ± 4.04	0.824
BMI (kg/m^2^)	24.99 ± 3.20	25.91 ± 3.58	0.081
FBG (mg/dL)	90.63 ± 9.61	90.89 ± 10.75	0.868
Insulin (μU/mL)	2.66 (1.95–3.77)	3.90 (2.89–5.72)	<0.001
HOMA-IR	0.58 (0.41–0.82)	0.82 (0.61–1.28)	<0.001
TG (mg/dL)	119.23 ± 38.77	122.49 ± 52.44	0.645
TC (mg/dL)	161.71 ± 39.94	172.19 ± 34.42	0.068
LDL-C (mg/dL)	95.82 ± 29.15	101.33 ± 26.30	0.196
HDL-C (mg/dL)	47.35 ± 7.09	44.93 ± 8.98	0.052
FSH (IU/L)	5.97 (4.82–8.34)	7.24 (5.23–10.10)	0.044
LH (IU/L)	8.28 (7.09–10.08)	17.42 (10.36–27.74)	<0.001
FT (pg/mL)	1.53 ± 0.33	3.13 ± 0.93	<0.001
LH/FSH ratio	1.29 (0.95–1.89)	2.24 (1.61–3.34)	<0.001

The data are presented as mean ± standard deviation (SD) or median and (interquartile range). PCOS: Polycystic ovarian syndrome; BMI: Body mass index; FBG: Fasting blood glucose; HOMA-IR: Homeostasis Model Assessment of Insulin Resistance; TG: Triglycerides; TC: Total cholesterol; LDL-C: Low density lipoprotein cholesterol; HDL-C: High density lipoprotein cholesterol; FSH: Follicle-stimulating hormone; LH: Luteinizing hormone; FT: Free testosterone. Independent Student’s t-test and Mann-Whitney U test were used to determine the differences of variables between two groups. p<0.05 was considered statistically significant.

**Fig 1:**
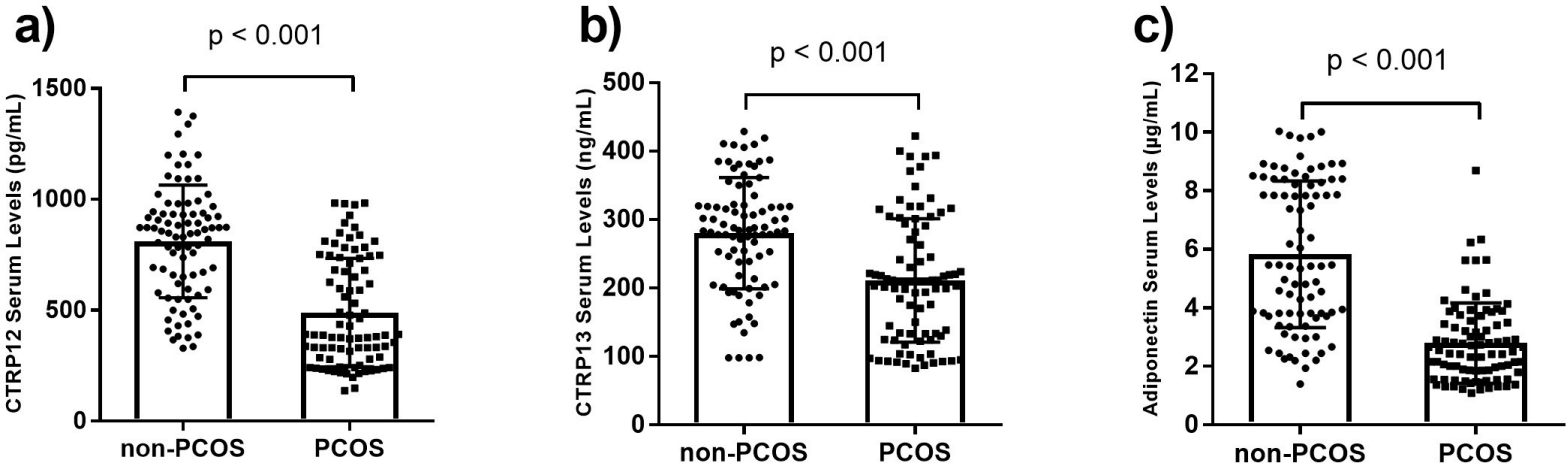
Serum concentrations of CTRP12 (a), CTRP13 (b), and adiponectin (c) in non-PCOS (n = 85) and PCOS (n = 86) groups. The data are presented as mean ± standard deviation (SD). PCOS: Polycystic ovarian syndrome. Independent Student’s t-test was used to determine the differences of the adipokines between two groups.

PCOS women had lower serum concentrations of adiponectin compared to non-PCOS women (p<0.001). Similarly, there were significantly lower CTRP12 and CTRP13 serum concentrations in PCOS women compared to non-PCOS women (p<0.001; Fig 1). Analysis of covariance (ANCOVA) was used to compare serum adiponectin, CTRP12, and CTRP13 levels between the non-PCOS and PCOS groups after adjustments for potential covariates of age, BMI, and HOMA-IR. Our findings showed that the difference of these three adipokines between non-PCOS and PCOS groups remained significant after adjustments (p<0.001).

**Fig 2:**
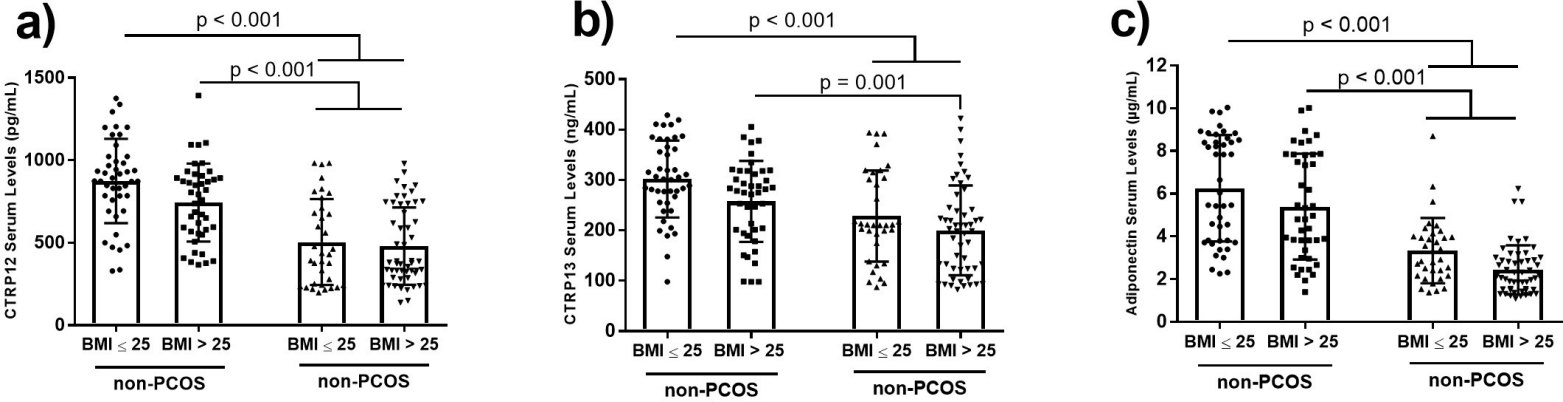
Serum concentrations of CTRP12 (a), CTRP13 (b), and adiponectin (c) in normal weight (BMI ≤25 kg/m^2^, n = 43) non-PCOS, overweight/obese (BMI >25 kg/m^2^, n = 42) non-PCOS, normal weight (BMI ≤25 kg/m^2^, n = 34) PCOS, and overweight/obese (BMI >25 kg/m^2^, n = 52) PCOS women. The data are presented as mean ± standard deviation (SD). ANOVA with Bonferroni corrections post hoc was used to determine the difference among the subgroups. PCOS: Polycystic ovarian syndrome.

We analyzed the data stratified by BMI (normal weight: BMI ≤25 kg/m^2^ and overweight/obese: BMI >25 kg/m^2^). According to the results, there were significantly lower serum concentrations of adiponectin, CTRP12, and CTRP13 in the overweight/obese and normal weight PCOS women compared to the corresponding non-PCOS subgroups despite the similar BMI values in the two subgroups. Serum levels of adiponectin and CTRP12 were significantly lower in normal weight PCOS women compared to obese non-PCOS women. However, no statistically significant difference in serum levels of adiponectin, CTRP12, and CTRP13 existed between the overweight/obese and normal weight subgroups in women with and without PCOS (Fig 2).

We observed a similar pattern in serum levels of the hormone profiles, which included LH, FSH, FT, and the LH/FSH ratio. Mean serum concentrations of LH and FT as well as the LH/FSH ratio differed between overweight/obese women with PCOS and those without PCOS. The overweight/obese women with PCOS had higher serum levels of LH, FT, and a higher LH/FSH ratio compared to overweight/obese women without PCOS. Normal weight women with PCOS had higher serum levels of FT, LH, FSH, and a higher LH/FSH ratio compared to both normal weight and overweight/obese women without PCOS. As with the adipokines, we did not find a significant difference in mean serum concentrations of LH and FT, and the LH/FSH ratio between normal weight women with PCOS and overweight/obese women with PCOS. However, there were lower FSH serum levels in overweight/obese women with PCOS compared to normal weight women with PCOS (Table 2).

**Table 2:** Clinical and laboratory parameters of overweight/obese and normal weight subjects with or without PCOS.

Parameters	Non-PCOS (n = 85)		PCOS (n = 86)		
	BMI ≤25 kg/m^2^ (n = 43)	BMI >25 kg/m^2^ (n = 42)	BMI ≤25 kg/m^2^ (n = 34)	BMI >25 kg/m^2^ (n = 52)	p-value
CTRP12 (pg/mL)	875.01 ± 255.61	744.50 ± 236.50	504.95 ± 260.64^‡^ ^#^	480.05 ± 234.45*	<0.001
CTRP13 (ng/mL)	302.08 ± 76.62	257.79 ± 80.30	228.55 ± 90.53^‡^	200.21 ± 89.10*	<0.001
BMI (kg/m^2^)	22.42 ± 1.74	27.63 ± 1.96	22.49 ± 1.72^#^	28.14 ± 2.57^†^	<0.001
FBG (mg/dL)	89.12 ± 7.83	92.19 ± 11.02	88.00 ± 6.53	92.79 ± 12.49	0.088
Insulin (μU/mL))	2.35 (1.55–3.15)	3.15 (2.13–4.64)	3.17 (2.17–4.04)	4.48 (3.36–6.89) * ^†^	<0.001
HOMA-IR	0.51 (0.31–0.68)	0.66 (0.47–1.18)	0.68 (0.47–0.89)	0.94 (0.67–1.65)* ^†^	<0.001
Adiponectin (μg/mL)	6.25 ± 2.49	5.39 ± 2.48	3.33 ± 1.53^‡^ ^#^	2.44 ± 1.15*	<0.001
FSH (IU/L)	5.860 (4.23–8.34)	6.62 (4.96–8.40)	7.95 (6.41–12.75)^‡^^#^	6.66 (4.39–9.07)^†^	0.001
LH (IU/L)	8.46 (7.29–10.17)	8.09 (6.85–9.82)	20.05 (12.98–30.75)^‡^^#^	16.31 (7.92–21.65)*	<0.001
FT (pg/mL)	1.55 ± 0.35	1.51 ± 0.30	3.05 ± 0.85^‡^^#^	3.18 ± 0.97*	<0.001
LH/FSH ratio	1.43 (0.94–1.86)	1.19 (0.95–1.89)	2.35 (1.74–3.44)^‡^^#^	2.10 (1.55–3.17)*	<0.001

The data are presented as mean ± standard deviation (SD) or median and (interquartile range). PCOS: Polycystic ovarian syndrome; BMI: Body mass index; FBG: Fasting blood glucose; HOMA-IR: Homeostasis Model Assessment of Insulin Resistance; FSH: Follicle-stimulating hormone; LH: Luteinizing hormone; FT: Free testosterone. ANOVA and Kruskal-Wallis with Bonferroni corrections post hoc were performed to determine the difference among the subgroups.

*p<0.05: Overweight/obese PCOS versus overweight/obese non-PCOS;

‡p<0.05: Normal weight PCOS versus normal weight non-PCOS;

†p<0.05: Normal weight PCOS versus overweight/obese PCOS;

^#^p<0.05: Normal weight PCOS versus overweight/obese non-PCOS.

Correlation analysis in the non-PCOS group revealed that adiponectin, CTRP12, and CTRP13 inversely correlated with BMI. CTRP12, but not adiponectin and CTRP13, was inversely correlated with log insulin and log HOMA-IR. Moreover, CTRP12 and CTRP13 were both correlated with adiponectin. Correlation analysis in the PCOS group showed that adiponectin, but not CTRP12 and CTRP13, had an inverse correlation with BMI. Adiponectin, CTRP12, and CTRP13 were inversely correlated with log insulin and log HOMA-IR. CTRP12 and CTRP13 were both correlated with adiponectin (Table 3).

**Table 3:** Correlations between adiponectin, CTRP12, and CTRP13 with anthropometric, hormonal and metabolic variables.

	Adiponectin		CTRP12		CTRP13	
	Non-PCOS	PCOS	Non-PCOS	PCOS	Non-PCOS	PCOS
CTRP12	0.360**	0.278**	1	1	0.220*	0.260*
CTRP13	0.241*	0.318**	0.220*	0.260*	1	1
Age	-0.014	-0.034	-0.121	0.136	-0.043	0.181
BMI	-0.231*	-0.364**	-0.310**	-0.075	-0.391**	-0.212
FBG	-0.241*	-0.236*	-0.156	-0.290**	-0.238*	0.065
Log insulin	-0.151	-0.304**	-0.254*	-0.230*	-0.071	-0.269*
Log HOMA-IR	-0.179	-0.319**	-0.258*	-0.266*	-0.105	-0.228*
TG	-0.140	-0.139	-0.078	-0.052	-0.134	0.049
TC	-0.097	-0.186	-0.058	-0.147	-0.097	-0.042
LDL-C	-0.035	-0.146	-0.129	-0.118	0.017	-0.093
HDL-C	0.038	-0.035	-0.007	-0.206	0.252*	-0.177
Adiponectin	1	1	0.360**	0.278**	0.241*	0.318**
Log FSH	0.074	0.137	-0.023	-0.063	-0.007	0.107
Log LH	0.007	0. 253*	-0.011	0.047	-0.040	0.229*
FT	-0.084	-0.162	0.013	-0.149	0.123	0.022
Log LH/FSH ratio	-0.056	0.235*	0.012	0.140	-0.018	0.230*

*p<0.05 and

**p<0.001

Pearson correlation analysis was performed to determine the relationships between variables. BMI: Body mass index; FBG: Fasting blood glucose; HOMA-IR: Homeostasis Model Assessment of Insulin Resistance; TG: Triglycerides; TC: Total cholesterol; LDL-C: Low density lipoprotein cholesterol; HDL-C: High density lipoprotein cholesterol; FSH: Follicle-stimulating hormone; LH: Luteinizing hormone; FT: Free testosterone

In the multiple linear regression model, we included variables that had significant correlations as independent variables to determine their association with serum levels of each adipokine. When adiponectin was included as the dependent variable and BMI, FBG, log insulin, log HOMA-IR, log LH, the log LH/FSH ratio, CTRP12, and CTRP13 were included as independent variables, we noted that the association of adiponectin with CTRP12 in the non-PCOS (B = 0.296; p = 0.009) remained significant. However, other variables lost their statistical significance. In a model that included CTRP12 as the dependent variable and BMI, FBG, log insulin, log HOMA-IR, adiponectin, and CTRP13 as independent variables, we observed that adiponectin (B = 0.287; p = 0.009) in the non-PCOS group and CTRP13 (B = 0.232, p = 0.041) in the PCOS group were significantly associated with CTRP12. Finally, in the model that used CTRP13 as the dependent variable and BMI, FBG, log insulin, log HOMA-IR, log LH, log LH/FSH ratio, adiponectin and CTRP12 as independent variables, we observed that BMI demonstrated a significant association with CTRP13 in the non-PCOS (B = -0.371, p = 0.001) and with CTRP12 in PCOS (B = 0.218, p = 0.047) groups.

## Discussion

A growing body of evidence suggests that adipokines play important roles in the pathogenesis of PCOS. Most adipokines are predominantly secreted by adipose tissue; hence, alterations in circulating levels of adipokines can mirror an aberrant function of adipose tissue [10–12].

Adiponectin is abundantly and exclusively expressed by adipose tissue [36]. Adiponectin enhances insulin sensitivity through various mechanisms [37]. Because it plays a protective role in PCOS, mainly through improvements to metabolic dysfunction, its minor beneficial effects on reproductive disturbances have been demonstrated [19]. Although the published data in alteration of adiponectin in PCOS is inconsistent [18], a substantial body of literature have reported reduced circulating levels of adiponectin in PCOS women compared to healthy individuals [16, 17, 38]. Consistently, in the current study, we observed lower levels of adiponectin in women with PCOS compared to those without PCOS. After we took into consideration the possible effect of obesity, we did not observe any significant difference in serum adiponectin levels between overweight/obese and normal weight women with or without PCOS. Correlation analysis showed an inverse correlation between adiponectin and BMI values in PCOS and non-PCOS groups, but it did not remain significant after adjustments for other variables in a multiple linear regression model. In contrast, previous studies reported a significant difference between obese and normal weight women in the PCOS group [11] or both PCOS and non-PCOS groups [13]. These discordant findings might be due to the differences in phenotypic distributions of PCOS patients and/or severity of obesity in study populations.

CTRP12 is an insulin sensitizing hormone predominantly expressed by adipose tissue [31]. An increasing amount of data largely derived from animal models has shown a role for CTRP12 in glucose homeostasis [31, 32]. CTRP12 improves glucose tolerance, attenuates inflammation, and reduces insulin resistance in diet-induced obese mice [31, 32]. In the present study we have found lower serum levels of CTRP12 in the PCOS group compared to the non-PCOS group after adjustments for age, BMI, and HOMA-IR. This finding supported the results of previous studies on PCOS and non-PCOS women, which reported lower serum concentrations of CTRP12 in the PCOS group compared to the non-PCOS group [24, 39]. Reduced CTRP12 expression has been observed in an animal model of obesity [32]. In our study, we did not observe any significant difference between overweight/obese and normal weight women with or without PCOS. Serum levels of CTRP12 had an inverse correlation with BMI in the non-PCOS group, but not in the PCOS group, which did not remain significant after adjustments in multiple linear regression. Consistently, whole population multiple regression analysis in former studies on PCOS and non-PCOS women did not show any significant association between CTRP12 and BMI. Several factors reportedly influence CTRP12 expression. Exposure of 3T3-L1 adipocytes to pro-inflammatory and endoplasmic reticulum stresses led to downregulation of CTRP12 expression suggestive of CTRP12 reduction in pathologic conditions related to adipose tissue dysfunction [32].

In humans, white adipose tissue is the main source of CTRP13 secretion [22]. CTRP13 can improve glucose metabolism and insulin sensitivity in normal weight and obese mice [22]. CTRP13 has anti-inflammatory effects postulated to be responsible for enhancing insulin sensitivity. After adjustments for age, HOMA-IR and BMI, our results have shown lower circulating levels of CTRP13 in the PCOS group compared to the non-PCOS group. There is no published data that addresses the role of CTRP13 in the pathogenesis of PCOS. However, numerous studies have reported lower circulating levels of CTRP13 in other metabolic disorders such as diabetes, non-alcoholic fatty liver, and coronary artery disease [27, 28]. We observed significantly lower serum levels of CTRP13 lower in both obese women with and without PCOS compared to the corresponding normal weight subgroups. This finding supported previous animal studies that reported elevated circulating levels of CTRP13 in obese mice [22]. In the current study, correlation and subsequent multiple linear regression analyses showed an association of CTRP13 and BMI that was independent of age, HOMA-IR, and BMI in non-PCOS group.

Collectively, we observed lower serum levels of adiponectin, CTRP12, and CTRP13 in PCOS group compared to non-PCOS group. Consistently, previous studies have reported lower circulating levels of CTRP3 and CTRP12 in PCOS [24, 25]. However, a recent study has indicated similar serum levels of CTRP9 in PCOS and control groups [23]. Accumulating evidence has demonstrated protective roles of these CTRPs in metabolic disorders [22, 31, 32, 40–42]. But, CTRP3, CTRP12, and CTRP13 may be regulated differently than CTRP9 in PCOS.

Moreover, our findings revealed a similar pattern of difference in adiponectin, CTRP12, and CTRP13 among overweight/obese and normal weight women with or without PCOS. While circulating levels of these three adipokines were found to be significantly lower in both overweight/obese and normal weight women with PCOS compared to their non-PCOS counterparts, there was no significant difference between overweight/obese and normal weight women with or without PCOS. Our findings supported previous studies’ results of adipose tissue dysfunction in both lean and obese women with PCOS [16]. In detail, they have indicated that adipose tissue of both lean and obese women with PCOS have aberrant morphology with enlarged adipocytes, and a different gene expression profile [16, 43–45]. These alterations, which are associated with hyperandrogenism, are presumed to contribute to metabolic disturbances in PCOS women. It is reasonable to postulate that adipose tissue secretory profiles can be altered in PCOS through mechanisms independent of obesity. Although one cannot rule out the role of obesity in determining serum concentrations of adipokines, even in pathologic conditions, but hormonal disturbances of PCOS patients, in particular hyperandrogenism, might mask the potential effects of obesity.

We recognize a number of limitations to our study. Due to the cross-sectional design of our study, it is not possible to deduce any causal relationships between the studied adipokines and other parameters. Moreover, the relatively small sample size of our study might affect the findings. Additionally, we did not consider the different phenotypes of PCOS which would be an assist to elucidate the possible roles of these adipokines in the pathogenesis of PCOS.

## Conclusions

We demonstrated lower circulating levels of adiponectin, CTRP12, and CTRP13 in PCOS patients compared to non-PCOS individuals. Our findings showed that serum levels of these adipokines did not differ between overweight/obese and normal weight women with or without PCOS. The associations of adiponectin, CTRP12, and CTRP13 with PCOS were not confounded by BMI. It seems that altered circulating levels of these adipokines are more related to other factors rather than obesity in PCOS condition. However, further studies are needed to improve our understanding about the role of adiponectin, CTRP12 and CTRP13 in PCOS and the influence of a possible interaction between PCOS and obesity on the circulating levels of these adipokines.

## Supporting information

S1 File Datasets supporting the conclusions of this article. (DOCX)
