# Supplementary material for: Lower circulating levels of CTRP12 and CTRP13 in polycystic ovarian syndrome: Irrespective of obesity
Source: PLoS One. 2018 Dec 12;13(12):e0208059. doi: 10.1371/journal.pone.0208059 (PMC6291267; doi:10.1371/journal.pone.0208059)
Supplement: S1 File — (DOCX) [file pone.0208059.s001.docx]

| group | BMI_Cat | adiponectin | age | ctrp12 | ctrp13 | BMI | FBG | insulin | HOMA_IR | TG | TC | LDL-C | HDL-C | FSH | LH | LH_FSH_ratio | FT |
| --- | --- | --- | --- | --- | --- | --- | --- | --- | --- | --- | --- | --- | --- | --- | --- | --- | --- |
| 1 | 3 | 4.89 | 29 | 1092.3 | 199.3 | 22.8 | 94 | 2.11 | 0.489728 | 134 | 185 | 96 | 42 | 3.71 | 7.67 | 2.067385 | 1.46 |
| 1 | 3 | 9.18 | 31 | 893.5 | 98.1 | 24.8 | 102 | 3.17 | 0.79837 | 112 | 154 | 105 | 45 | 8.16 | 10.27 | 1.258578 | 1.15 |
| 1 | 3 | 4.58 | 31 | 938.5 | 148.2 | 21.3 | 97 | 1.92 | 0.459852 | 139 | 164 | 95 | 46 | 7.64 | 6.68 | 0.874346 | 1.9 |
| 1 | 3 | 9.8 | 34 | 872.4 | 381.2 | 21.5 | 89 | 2.61 | 0.573556 | 143 | 164 | 85 | 53 | 11.81 | 9.13 | 0.773074 | 1.26 |
| 1 | 3 | 5.46 | 34 | 872.9 | 278.1 | 24.5 | 95 | 4.1 | 0.961728 | 130 | 172 | 91 | 48 | 4.82 | 8.64 | 1.792531 | 1.36 |
| 1 | 3 | 8.21 | 34 | 848.2 | 384.9 | 25 | 87 | 3.11 | 0.668074 | 128 | 158 | 73 | 55 | 3.76 | 10.91 | 2.901596 | 1.39 |
| 1 | 3 | 10.04 | 33 | 1203.9 | 411 | 21.7 | 88 | 1.44 | 0.312889 | 117 | 143 | 79 | 53 | 6.84 | 5.39 | 0.788012 | 1.81 |
| 1 | 3 | 8.39 | 23 | 1022.9 | 267.1 | 20.2 | 94 | 2.89 | 0.670765 | 157 | 179 | 89 | 48 | 5.86 | 9.38 | 1.600683 | 1.13 |
| 1 | 3 | 5.42 | 28 | 874.9 | 193.5 | 22.4 | 79 | 0.89 | 0.173605 | 89 | 154 | 76 | 57 | 5.91 | 7.26 | 1.228426 | 1.48 |
| 1 | 3 | 3.81 | 27 | 473.1 | 254.3 | 23.4 | 94 | 3.23 | 0.749679 | 120 | 155 | 88 | 53 | 13.12 | 8.34 | 0.635671 | 2.31 |
| 1 | 3 | 3.37 | 37 | 336.9 | 361.4 | 21.8 | 82 | 1.34 | 0.271309 | 37 | 164 | 109 | 62 | 5.59 | 9.13 | 1.633274 | 1.82 |
| 1 | 3 | 2.44 | 33 | 500.2 | 319.4 | 22.8 | 76 | 2.19 | 0.410963 | 165 | 204 | 118 | 53 | 4.97 | 8.28 | 1.665996 | 1.15 |
| 1 | 3 | 3.1 | 31 | 482.9 | 218.2 | 24.4 | 87 | 2.93 | 0.629407 | 151 | 172 | 95 | 48 | 7.81 | 6.28 | 0.804097 | 2.08 |
| 1 | 3 | 8.92 | 36 | 934.6 | 409.2 | 22.7 | 92 | 2.35 | 0.533827 | 93 | 143 | 69 | 52 | 3.3 | 4.28 | 1.29697 | 1.37 |
| 1 | 3 | 7.84 | 26 | 830.1 | 387.2 | 17.2 | 81 | 1.55 | 0.31 | 46 | 134 | 84 | 41 | 5.86 | 7.67 | 1.308874 | 2.18 |
| 1 | 3 | 3 | 28 | 1200.3 | 429.1 | 22.9 | 93 | 1.95 | 0.447778 | 92 | 130 | 92 | 43 | 5.65 | 10.84 | 1.918584 | 2.01 |
| 1 | 3 | 8.52 | 30 | 873.5 | 419.3 | 20.8 | 75 | 2.56 | 0.474074 | 81 | 137 | 70 | 51 | 6.57 | 6.57 | 1 | 1.74 |
| 1 | 3 | 8.84 | 36 | 831.1 | 310.9 | 21.3 | 92 | 1.04 | 0.236247 | 104 | 87 | 42 | 52 | 4.18 | 10.27 | 2.456938 | 1.17 |
| 1 | 3 | 5.46 | 36 | 693.5 | 239.1 | 24.1 | 89 | 4.19 | 0.920765 | 187 | 176 | 132 | 39 | 2.59 | 8.46 | 3.266409 | 1.79 |
| 1 | 3 | 7.84 | 32 | 758.8 | 283.4 | 19.3 | 78 | 1.55 | 0.298519 | 88 | 92 | 65 | 71 | 3.39 | 10.84 | 3.19764 | 2.17 |
| 1 | 3 | 2.25 | 30 | 967.7 | 289.1 | 22.3 | 76 | 0.84 | 0.15763 | 84 | 130 | 92 | 63 | 5.86 | 7.97 | 1.360068 | 1.71 |
| 1 | 3 | 7.83 | 32 | 944.2 | 277.1 | 23.2 | 82 | 2.35 | 0.475802 | 107 | 210 | 102 | 48 | 4.23 | 7.32 | 1.730496 | 1.48 |
| 1 | 3 | 2.31 | 24 | 846.1 | 320.1 | 24.1 | 87 | 0.92 | 0.19763 | 78 | 201 | 134 | 48 | 6.02 | 9.32 | 1.548173 | 1.39 |
| 1 | 3 | 4.46 | 34 | 691.3 | 301.9 | 23.4 | 104 | 6.33 | 1.625481 | 68 | 159 | 121 | 53 | 3.85 | 13.03 | 3.384416 | 1.75 |
| 1 | 3 | 8.92 | 21 | 1199.4 | 365.5 | 19.2 | 92 | 2.41 | 0.547457 | 111 | 132 | 75 | 45 | 11.62 | 7.97 | 0.685886 | 1.61 |
| 1 | 3 | 3.39 | 27 | 784.4 | 205 | 24 | 86 | 2.9 | 0.615802 | 151 | 182 | 105 | 37 | 10.03 | 13.91 | 1.386839 | 1.08 |
| 1 | 3 | 3.81 | 25 | 329.5 | 315.5 | 24.6 | 94 | 7.52 | 1.745383 | 155 | 127 | 75 | 48 | 3.3 | 10.20 | 3.090909 | 1 |
| 1 | 3 | 8.4 | 22 | 922.5 | 305.6 | 23.1 | 86 | 6.77 | 1.43758 | 187 | 176 | 132 | 39 | 2.71 | 7.67 | 2.830258 | 1.98 |
| 1 | 3 | 3.65 | 23 | 456.8 | 410 | 20.7 | 109 | 5.13 | 1.380667 | 130 | 179 | 120 | 57 | 8.69 | 8.28 | 0.952819 | 1.46 |
| 1 | 3 | 8.29 | 32 | 1155.3 | 278.1 | 24.2 | 85 | 0.88 | 0.184691 | 85 | 152 | 90 | 48 | 9.36 | 10.08 | 1.076923 | 1.65 |
| 1 | 3 | 3.79 | 31 | 549.3 | 306.7 | 22.8 | 95 | 2.19 | 0.513704 | 144 | 145 | 102 | 50 | 5.49 | 3.72 | 0.677596 | 1.75 |
| 1 | 3 | 3.71 | 25 | 785.4 | 322.1 | 24.1 | 87 | 3.15 | 0.676667 | 134 | 132 | 51 | 42 | 8.34 | 5.83 | 0.699041 | 2.16 |
| 1 | 3 | 8.4 | 27 | 918.3 | 189.3 | 23.8 | 84 | 1.44 | 0.298667 | 125 | 225 | 126 | 51 | 10.28 | 8.70 | 0.846304 | 1.88 |
| 1 | 3 | 5.42 | 27 | 659.1 | 255.7 | 22.1 | 93 | 3.15 | 0.723333 | 92 | 187 | 98 | 43 | 4.52 | 7.26 | 1.606195 | 1.24 |
| 1 | 3 | 8.19 | 25 | 1339.5 | 350 | 21.4 | 84 | 2.85 | 0.591111 | 146 | 100 | 45 | 42 | 4.97 | 11.04 | 2.221328 | 1.37 |
| 1 | 3 | 8.83 | 27 | 931 | 281.5 | 24.7 | 86 | 2.1 | 0.445926 | 132 | 149 | 102 | 37 | 5.65 | 8.82 | 1.561062 | 1.01 |
| 1 | 3 | 4.56 | 22 | 1375 | 356 | 21.3 | 88 | 2.14 | 0.464988 | 167 | 94 | 45 | 45 | 10.1 | 11.63 | 1.151485 | 1.55 |
| 1 | 3 | 8.78 | 31 | 1022.6 | 381.3 | 22.1 | 96 | 0.94 | 0.222815 | 61 | 210 | 163 | 46 | 4.28 | 9.89 | 2.310748 | 1.49 |
| 1 | 3 | 3.71 | 31 | 1294.2 | 284.3 | 21.2 | 92 | 2.39 | 0.542914 | 66 | 186 | 109 | 48 | 3.22 | 4.59 | 1.425466 | 1.61 |
| 1 | 3 | 6.64 | 28 | 1156.4 | 278.1 | 21.3 | 78 | 2.05 | 0.394815 | 103 | 104 | 59 | 50 | 8.4 | 6.34 | 0.754762 | 1.18 |
| 1 | 3 | 6.04 | 23 | 983.5 | 300.6 | 20.4 | 95 | 2.47 | 0.579383 | 70 | 189 | 131 | 50 | 7.99 | 14.39 | 1.801001 | 1.14 |
| 1 | 3 | 8.6 | 28 | 785.9 | 238.1 | 24.3 | 104 | 3.21 | 0.824296 | 141 | 159 | 104 | 44 | 4.97 | 8.21 | 1.651911 | 1.29 |
| 1 | 3 | 9.85 | 30 | 993.5 | 385.1 | 21 | 85 | 2.13 | 0.447037 | 107 | 210 | 120 | 43 | 10.98 | 10.14 | 0.923497 | 1.34 |
| 1 | 4 | 2.44 | 31 | 1392.9 | 98.3 | 27.5 | 99 | 3.38 | 0.826222 | 154 | 210 | 112 | 41 | 5.54 | 6.85 | 1.236462 | 1.11 |
| 1 | 4 | 7.83 | 32 | 980.2 | 281.9 | 25.5 | 93 | 2.11 | 0.484519 | 142 | 174 | 95 | 44 | 4.97 | 6.45 | 1.297787 | 1.2 |
| 1 | 4 | 7.38 | 31 | 568.1 | 150.6 | 25.6 | 95 | 1.5 | 0.351852 | 106 | 144 | 74 | 47 | 5.13 | 10.72 | 2.089669 | 1.79 |
| 1 | 4 | 2.99 | 37 | 793 | 378.1 | 25.2 | 103 | 5.12 | 1.302123 | 162 | 241 | 114 | 38 | 8.4 | 10.84 | 1.290476 | 1.66 |
| 1 | 4 | 6.18 | 40 | 917.2 | 178.4 | 30.4 | 105 | 4.18 | 1.083704 | 183 | 254 | 120 | 42 | 4.87 | 9.38 | 1.926078 | 1.48 |
| 1 | 4 | 10.02 | 20 | 893.4 | 271.3 | 29 | 88 | 2.94 | 0.638815 | 166 | 186 | 143 | 41 | 8.34 | 7.97 | 0.955635 | 1.17 |
| 1 | 4 | 4.81 | 35 | 578.9 | 147.3 | 29.1 | 106 | 5.6 | 1.465679 | 177 | 216 | 110 | 38 | 5.81 | 5.50 | 0.946644 | 1.06 |
| 1 | 4 | 3.81 | 27 | 389.5 | 157.9 | 26.4 | 97 | 1.28 | 0.306568 | 147 | 170 | 103 | 45 | 9.11 | 11.76 | 1.290889 | 1.11 |
| 1 | 4 | 4.36 | 27 | 377.4 | 201.3 | 26 | 97 | 0.93 | 0.222741 | 99 | 164 | 106 | 38 | 7.24 | 8.15 | 1.125691 | 1.69 |
| 1 | 4 | 3.81 | 33 | 596.1 | 318.2 | 25.2 | 96 | 3.87 | 0.917333 | 102 | 188 | 92 | 47 | 3.09 | 7.62 | 2.466019 | 0.81 |
| 1 | 4 | 2.19 | 37 | 620.1 | 285.4 | 27.5 | 104 | 7.03 | 1.805235 | 169 | 254 | 134 | 38 | 3.3 | 6.34 | 1.921212 | 1.4 |
| 1 | 4 | 8.47 | 31 | 506.3 | 335 | 25.7 | 76 | 1.78 | 0.334025 | 101 | 177 | 116 | 54 | 6.62 | 3.82 | 0.577039 | 1.5 |
| 1 | 4 | 3.88 | 35 | 549.2 | 278.4 | 30.8 | 76 | 3.22 | 0.604247 | 93 | 129 | 75 | 52 | 6.07 | 7.79 | 1.283361 | 1.28 |
| 1 | 4 | 5.42 | 33 | 439.1 | 98.4 | 30.4 | 82 | 2.87 | 0.581086 | 102 | 138 | 77 | 41 | 4.42 | 9.70 | 2.19457 | 1.48 |
| 1 | 4 | 2.2 | 31 | 738.2 | 98.2 | 29.7 | 106 | 2.19 | 0.573185 | 69 | 154 | 21 | 53 | 7.12 | 6.11 | 0.858146 | 1.78 |
| 1 | 4 | 2.54 | 31 | 849 | 318.2 | 30.5 | 108 | 4.55 | 1.213333 | 110 | 106 | 94 | 44 | 5.44 | 4.59 | 0.84375 | 2.28 |
| 1 | 4 | 4.28 | 32 | 857.3 | 301.4 | 28.7 | 72 | 1.89 | 0.336 | 97 | 198 | 136 | 43 | 2.59 | 9.51 | 3.671815 | 1.62 |
| 1 | 4 | 8.94 | 33 | 882.5 | 352.1 | 25.8 | 74 | 3.58 | 0.654123 | 68 | 166 | 110 | 42 | 7.7 | 6.85 | 0.88961 | 1.89 |
| 1 | 4 | 1.39 | 27 | 406.6 | 204.3 | 27.7 | 97 | 4.92 | 1.17837 | 151 | 182 | 105 | 37 | 6.62 | 11.82 | 1.785498 | 1.37 |
| 1 | 4 | 8.39 | 35 | 873.4 | 375.2 | 27.9 | 75 | 3.14 | 0.581481 | 172 | 198 | 149 | 42 | 9.24 | 10.59 | 1.146104 | 1.55 |
| 1 | 4 | 3.82 | 30 | 983 | 134.9 | 29.7 | 107 | 8.02 | 2.118864 | 155 | 127 | 75 | 48 | 7.81 | 7.56 | 0.96799 | 2.02 |
| 1 | 4 | 6.39 | 27 | 650.7 | 253.1 | 26.2 | 97 | 3.15 | 0.754444 | 85 | 152 | 90 | 48 | 5.7 | 4.28 | 0.750877 | 1.52 |
| 1 | 4 | 7.48 | 33 | 758.9 | 275.3 | 25.7 | 96 | 4.1 | 0.971852 | 93.8 | 213.3 | 142 | 52.6 | 9.05 | 4.07 | 0.449724 | 1.7 |
| 1 | 4 | 2.66 | 34 | 1093.3 | 318.5 | 25.3 | 89 | 1.34 | 0.294469 | 187 | 165 | 78 | 48 | 3.62 | 9.32 | 2.574586 | 1.48 |
| 1 | 4 | 4.96 | 32 | 805.6 | 293.1 | 30.1 | 82 | 6.24 | 1.263407 | 93 | 125 | 72 | 68 | 6.9 | 8.89 | 1.288406 | 1.8 |
| 1 | 4 | 4.81 | 31 | 556.2 | 213.4 | 27.9 | 86 | 3.11 | 0.660395 | 81 | 91 | 64 | 43 | 3.8 | 9.51 | 2.502632 | 1.85 |
| 1 | 4 | 3.94 | 31 | 933.4 | 405.6 | 27 | 98 | 2.54 | 0.614617 | 107 | 178 | 91 | 68 | 7.52 | 7.67 | 1.019947 | 2.09 |
| 1 | 4 | 5.33 | 32 | 383.5 | 194.3 | 28.5 | 79 | 3.95 | 0.770494 | 99 | 94 | 73 | 44 | 4.13 | 10.08 | 2.440678 | 1.63 |
| 1 | 4 | 7.85 | 33 | 659 | 189.4 | 27.8 | 100 | 6.87 | 1.696296 | 143 | 188 | 102 | 37 | 9.72 | 9.95 | 1.023663 | 1.39 |
| 1 | 4 | 7.81 | 33 | 672.2 | 288.1 | 28.9 | 106 | 2.15 | 0.562716 | 125 | 89 | 54 | 52 | 8.4 | 13.23 | 1.575 | 1.69 |
| 1 | 4 | 1.93 | 34 | 593.5 | 246.5 | 29.3 | 93 | 1.44 | 0.330667 | 59 | 98 | 62 | 53 | 10.34 | 11.43 | 1.105416 | 1.3 |
| 1 | 4 | 3.84 | 25 | 431.2 | 288.3 | 29.3 | 109 | 8.42 | 2.266123 | 172 | 198 | 149 | 42 | 11.87 | 6.91 | 0.58214 | 1.05 |
| 1 | 4 | 8.74 | 31 | 1105.4 | 321.1 | 26.8 | 93 | 5.15 | 1.182593 | 104 | 87 | 42 | 52 | 4.62 | 9.82 | 2.125541 | 1.1 |
| 1 | 4 | 2.44 | 26 | 685.2 | 201.5 | 25.4 | 85 | 3.15 | 0.661111 | 156 | 210 | 165 | 41 | 8.1 | 9.07 | 1.119753 | 1.68 |
| 1 | 4 | 7.83 | 29 | 820.5 | 283.5 | 27.8 | 98 | 3.77 | 0.912247 | 125 | 235 | 134 | 51 | 12.46 | 8.28 | 0.664526 | 1.58 |
| 1 | 4 | 9.89 | 30 | 1094.2 | 311.8 | 32 | 73 | 0.76 | 0.136988 | 57 | 189 | 132 | 54 | 10.28 | 8.03 | 0.781128 | 1.78 |
| 1 | 4 | 5.46 | 24 | 906.4 | 246.7 | 25.4 | 85 | 0.55 | 0.115432 | 42 | 172 | 89 | 48 | 4.92 | 5.72 | 1.162602 | 1.35 |
| 1 | 4 | 8.48 | 26 | 870.4 | 253.1 | 25.6 | 89 | 2.66 | 0.584543 | 108 | 98 | 56 | 46 | 7.01 | 7.09 | 1.011412 | 1.33 |
| 1 | 4 | 7.33 | 27 | 893.5 | 275.4 | 25.8 | 84 | 3.19 | 0.66163 | 113 | 153 | 78 | 45 | 6.62 | 12.49 | 1.886707 | 1.48 |
| 1 | 4 | 3.35 | 20 | 864.7 | 299.1 | 25.6 | 79 | 2.14 | 0.417432 | 143 | 142 | 87 | 38 | 5.97 | 7.32 | 1.226131 | 1.28 |
| 1 | 4 | 2.95 | 25 | 367.3 | 319.4 | 30.2 | 106 | 6.73 | 1.761432 | 231 | 168 | 103 | 45 | 13.12 | 8.21 | 0.625762 | 1.48 |
| 1 | 4 | 7.86 | 27 | 932.6 | 385.1 | 25.5 | 89 | 2.45 | 0.538395 | 129 | 119 | 67 | 45 | 5.54 | 7.62 | 1.375451 | 1.71 |
| 2 | 1 | 4.61 | 39 | 983.1 | 319.4 | 23.23 | 81 | 1.78 | 0.356 | 301 | 207 | 97 | 39 | 10.8 | 23.75 | 2.199074 | 2.57 |
| 2 | 1 | 6.33 | 36 | 975.6 | 315.3 | 23.24 | 84 | 2.07 | 0.429333 | 84 | 131 | 75 | 30 | 8.6 | 17.37 | 2.019767 | 2.63 |
| 2 | 1 | 2.78 | 27 | 429.1 | 211.4 | 23.44 | 85 | 3.25 | 0.682099 | 60 | 141 | 99 | 30 | 2.3 | 7.33 | 3.186957 | 3.29 |
| 2 | 1 | 8.7 | 26 | 251.5 | 291.1 | 21.97 | 93 | 2.67 | 0.613111 | 116 | 137 | 66 | 36 | 25.6 | 63.26 | 2.471094 | 2.95 |
| 2 | 1 | 1.79 | 28 | 241.8 | 329 | 22.31 | 87 | 2.91 | 0.625111 | 124 | 150 | 70 | 52 | 24.1 | 23.13 | 0.959751 | 2.98 |
| 2 | 1 | 3.72 | 27 | 377.4 | 134.2 | 23.78 | 84 | 6.52 | 1.352296 | 117 | 217 | 148 | 46 | 15 | 11.1 | 0.74 | 2 |
| 2 | 1 | 2.71 | 28 | 894.1 | 95 | 18.29 | 94 | 2.05 | 0.475802 | 101 | 148 | 81 | 35 | 10.3 | 24.49 | 2.37767 | 2.45 |
| 2 | 1 | 1.53 | 30 | 229.4 | 392.1 | 19.33 | 86 | 2 | 0.424691 | 119 | 129 | 60 | 45 | 16.9 | 20.39 | 1.206509 | 1.78 |
| 2 | 1 | 2.01 | 33 | 478.5 | 210.4 | 21.16 | 94 | 3.07 | 0.712543 | 97 | 174 | 104 | 51 | 5.25 | 10.48 | 1.99619 | 3.19 |
| 2 | 1 | 2.54 | 30 | 802.1 | 213.4 | 23.44 | 74 | 2.64 | 0.48237 | 86 | 167 | 105 | 42 | 12.9 | 77.93 | 6.041085 | 3.25 |
| 2 | 1 | 4.14 | 31 | 650.3 | 392.1 | 24.04 | 88 | 11 | 2.390123 | 282 | 265 | 168 | 41 | 7 | 28.71 | 4.101429 | 1.7 |
| 2 | 1 | 4.25 | 30 | 224.5 | 263.3 | 18.67 | 90 | 2.05 | 0.455556 | 47 | 123 | 55 | 47 | 6.74 | 15.66 | 2.323442 | 1.92 |
| 2 | 1 | 1.37 | 29 | 215.5 | 97.3 | 23.74 | 92 | 6.73 | 1.52879 | 109 | 145 | 82 | 41 | 12.7 | 32.33 | 2.545669 | 3.5 |
| 2 | 1 | 3.92 | 34 | 983.2 | 371.1 | 22.03 | 91 | 4.29 | 0.963926 | 122 | 167 | 106 | 37 | 6.7 | 23.06 | 3.441791 | 3.69 |
| 2 | 1 | 1.44 | 38 | 561.2 | 174.6 | 24.8 | 88 | 3.29 | 0.714864 | 97 | 177 | 103 | 61 | 13.7 | 12.98 | 0.947445 | 3.81 |
| 2 | 1 | 3.32 | 23 | 228.5 | 117.2 | 22.94 | 87 | 2.67 | 0.573556 | 41 | 127 | 53 | 52 | 4.6 | 12.87 | 2.797826 | 4.07 |
| 2 | 1 | 4.51 | 27 | 198.5 | 138.5 | 18.67 | 84 | 1.86 | 0.385778 | 71 | 157 | 73 | 72 | 6.9 | 14.69 | 2.128986 | 4.42 |
| 2 | 1 | 2.41 | 31 | 332.5 | 217.4 | 20.7 | 86 | 2.21 | 0.469284 | 62 | 143 | 79 | 50 | 8.1 | 14.07 | 1.737037 | 2.68 |
| 2 | 1 | 2.41 | 27 | 235.1 | 203.3 | 23.31 | 89 | 2.94 | 0.646074 | 76 | 217 | 147 | 55 | 10.5 | 27.74 | 2.641905 | 1.75 |
| 2 | 1 | 2.51 | 23 | 812.3 | 201.4 | 23.44 | 85 | 3.95 | 0.829012 | 91 | 183 | 126 | 39 | 18.44 | 40.99 | 2.222885 | 3.63 |
| 2 | 1 | 3.85 | 34 | 680.2 | 193 | 22.48 | 83 | 4.28 | 0.877136 | 72 | 154 | 101.6 | 38 | 4.2 | 9.16 | 2.180952 | 3.97 |
| 2 | 1 | 1.55 | 31 | 462.7 | 103.9 | 24.17 | 88 | 3.3 | 0.717037 | 127 | 175 | 104 | 39.8 | 3.86 | 5.18 | 1.341969 | 2 |
| 2 | 1 | 2.01 | 31 | 377.1 | 210.4 | 23.34 | 83 | 3.83 | 0.784914 | 122 | 150 | 67 | 43 | 15.8 | 55.6 | 3.518987 | 4.95 |
| 2 | 1 | 2.83 | 26 | 244.1 | 199.2 | 21.08 | 83 | 3.11 | 0.637358 | 89 | 172 | 83 | 55 | 7.02 | 30.75 | 4.380342 | 2.71 |
| 2 | 1 | 3.55 | 34 | 773.8 | 319.4 | 24.1 | 91 | 3.85 | 0.865062 | 103 | 188 | 104 | 46 | 5.53 | 13.49 | 2.439421 | 3.13 |
| 2 | 1 | 2.15 | 32 | 391.3 | 211.6 | 23.03 | 92 | 2.01 | 0.456593 | 69 | 142 | 88 | 61 | 8.31 | 14.06 | 1.691937 | 4.55 |
| 2 | 1 | 3.92 | 26 | 827.3 | 210.3 | 20.98 | 71 | 2.01 | 0.35237 | 67 | 150 | 78 | 37 | 7.65 | 17.36 | 2.269281 | 2.83 |
| 2 | 1 | 3.96 | 28 | 388.1 | 218.3 | 24.12 | 88 | 3.23 | 0.701827 | 113 | 143 | 92 | 41 | 5.33 | 19.72 | 3.699812 | 2.74 |
| 2 | 1 | 2.15 | 30 | 278.8 | 394.1 | 22.1 | 93 | 3.99 | 0.916222 | 83 | 154 | 76 | 45 | 8.37 | 32.18 | 3.844683 | 3.88 |
| 2 | 1 | 3.99 | 35 | 219.4 | 304.5 | 24.33 | 101 | 4.2 | 1.047407 | 109 | 221 | 138 | 58 | 8.09 | 31.08 | 3.84178 | 3.01 |
| 2 | 1 | 3.4 | 31 | 598.1 | 199.2 | 23.4 | 83 | 3.31 | 0.678346 | 189 | 197 | 136 | 44 | 7.27 | 21.39 | 2.942228 | 4.07 |
| 2 | 1 | 5.62 | 26 | 711.8 | 301.5 | 23.51 | 93 | 2.89 | 0.66363 | 100 | 88 | 67 | 41 | 7.81 | 32.66 | 4.181818 | 2.08 |
| 2 | 1 | 4.38 | 24 | 674.3 | 130.3 | 22.1 | 98 | 4.81 | 1.163901 | 168 | 201 | 132 | 49 | 7.54 | 11.99 | 1.590186 | 2.36 |
| 2 | 1 | 2.91 | 26 | 437.2 | 87.4 | 23.41 | 103 | 4.93 | 1.253802 | 167 | 273 | 178 | 39 | 5.23 | 5.04 | 0.963671 | 3.29 |
| 2 | 2 | 1.22 | 30 | 812.3 | 219.5 | 31.24 | 97 | 9.39 | 2.248963 | 221 | 186 | 105 | 37 | 5.6 | 6.98 | 1.246429 | 3.66 |
| 2 | 2 | 1.86 | 37 | 839.1 | 133.1 | 28.13 | 81 | 3.66 | 0.732 | 140 | 155 | 85 | 42 | 2.43 | 2.34 | 0.962963 | 2.51 |
| 2 | 2 | 5.63 | 32 | 784.2 | 422.1 | 26.03 | 84 | 3 | 0.622222 | 112 | 206 | 112 | 46 | 3.92 | 20.9 | 5.331633 | 3.78 |
| 2 | 2 | 3.49 | 36 | 315 | 293.8 | 25.53 | 92 | 2.36 | 0.536099 | 100 | 191 | 107 | 38 | 8.4 | 37.47 | 4.460714 | 5.08 |
| 2 | 2 | 5.62 | 34 | 849.5 | 377.1 | 25.65 | 89 | 2.54 | 0.558173 | 110 | 133 | 60 | 51 | 7 | 35.71 | 5.101429 | 3.19 |
| 2 | 2 | 1.52 | 28 | 241.4 | 93.4 | 25.71 | 101 | 11.2 | 2.793086 | 242 | 204 | 119 | 37 | 7.4 | 10.06 | 1.359459 | 3.56 |
| 2 | 2 | 1.5 | 30 | 332.5 | 133.5 | 37.32 | 126 | 12.76 | 3.969778 | 301 | 236 | 128 | 33 | 7.2 | 17.85 | 2.479167 | 2.05 |
| 2 | 2 | 3.01 | 27 | 748 | 224.1 | 25.86 | 80 | 3.27 | 0.645926 | 80 | 160 | 98 | 39 | 5.1 | 5.98 | 1.172549 | 4.01 |
| 2 | 2 | 2.73 | 27 | 590.1 | 210.4 | 29.9 | 75 | 4.34 | 0.803704 | 63 | 144 | 70.4 | 61 | 9 | 10.45 | 1.161111 | 2.95 |
| 2 | 2 | 3.89 | 32 | 233.1 | 198.4 | 29.21 | 78 | 6.36 | 1.224889 | 144 | 153 | 98 | 46 | 2.6 | 3.07 | 1.180769 | 3.72 |
| 2 | 2 | 2.87 | 31 | 746.1 | 93.8 | 25.1 | 93 | 9.2 | 2.112593 | 97 | 159 | 84 | 42 | 4.3 | 17.58 | 4.088372 | 2.89 |
| 2 | 2 | 6.23 | 36 | 241.5 | 122.4 | 30.39 | 83 | 7.17 | 1.469407 | 116 | 166 | 94 | 55 | 16.1 | 19.58 | 1.216149 | 2.39 |
| 2 | 2 | 3.44 | 26 | 587.5 | 170.3 | 27.92 | 89 | 4.28 | 0.940543 | 124 | 179 | 112 | 43 | 10.2 | 29.82 | 2.923529 | 2.14 |
| 2 | 2 | 3.01 | 32 | 928.3 | 241.4 | 28.51 | 93 | 3.75 | 0.861111 | 82 | 180 | 119.6 | 44 | 3.58 | 7.42 | 2.072626 | 2.48 |
| 2 | 2 | 1.2 | 29 | 331.5 | 348.5 | 26.67 | 122 | 1.44 | 0.433778 | 218 | 209 | 133 | 36 | 4.5 | 7.59 | 1.686667 | 2.6 |
| 2 | 2 | 1.55 | 34 | 356.1 | 102.6 | 34.05 | 111 | 9.61 | 2.633852 | 126 | 121 | 78 | 62 | 4.1 | 6.29 | 1.534146 | 3.1 |
| 2 | 2 | 2.81 | 31 | 748.5 | 124.3 | 31.98 | 75 | 5.29 | 0.97963 | 104 | 104 | 79 | 62 | 2.1 | 5.08 | 2.419048 | 1.62 |
| 2 | 2 | 3.22 | 28 | 371.7 | 230.1 | 25.46 | 84 | 3.56 | 0.73837 | 79 | 162 | 100 | 49 | 4.07 | 14.42 | 3.542998 | 1.83 |
| 2 | 2 | 2.01 | 29 | 239.4 | 203.3 | 28.62 | 95 | 5.72 | 1.341728 | 136 | 201 | 125 | 49 | 8.21 | 17.48 | 2.129111 | 3.63 |
| 2 | 2 | 1.54 | 31 | 391.4 | 150.2 | 30.85 | 85 | 4.34 | 0.910864 | 77 | 200 | 130 | 55 | 8.12 | 15.53 | 1.912562 | 2.25 |
| 2 | 2 | 2.43 | 36 | 981.1 | 400.3 | 27.12 | 97 | 7.04 | 1.686123 | 131 | 184 | 88 | 50 | 6.1 | 16.57 | 2.716393 | 3.07 |
| 2 | 2 | 2.31 | 33 | 782.9 | 198.8 | 25.85 | 80 | 4.68 | 0.924444 | 163 | 166 | 92 | 34 | 20.3 | 54.52 | 2.685714 | 3.75 |
| 2 | 2 | 2.65 | 25 | 381.3 | 82.9 | 27.67 | 97 | 3.95 | 0.946049 | 146 | 178 | 109 | 44 | 11.7 | 19.84 | 1.695726 | 1.46 |
| 2 | 2 | 1.25 | 27 | 287.4 | 90.8 | 31.83 | 73 | 4.34 | 0.782272 | 99 | 144 | 85 | 39 | 5.6 | 16.05 | 2.866071 | 5.01 |
| 2 | 2 | 1.31 | 32 | 733.8 | 144.8 | 25.39 | 83 | 3.07 | 0.62916 | 68 | 169 | 88 | 48 | 4.09 | 8.26 | 2.01956 | 3.29 |
| 2 | 2 | 2.65 | 31 | 531.3 | 176 | 29.76 | 100 | 8.1 | 2 | 79 | 174 | 108 | 43 | 18 | 54.11 | 3.006111 | 3.13 |
| 2 | 2 | 2.15 | 35 | 138.3 | 302.5 | 28.3 | 114 | 11.55 | 3.251111 | 208 | 187 | 122 | 32 | 11.3 | 22.16 | 1.961062 | 3.47 |
| 2 | 2 | 1.36 | 26 | 377.5 | 211.3 | 27.06 | 93 | 3.78 | 0.868 | 142 | 110 | 48 | 34 | 10.1 | 18.56 | 1.837624 | 2.51 |
| 2 | 2 | 1.89 | 33 | 388.4 | 210.1 | 28.65 | 85 | 4.63 | 0.971728 | 111 | 192 | 122 | 48 | 6.9 | 12.3 | 1.782609 | 2.74 |
| 2 | 2 | 1.49 | 30 | 338.1 | 98.1 | 30.49 | 91 | 5.39 | 1.211086 | 142 | 187 | 105 | 37 | 8.19 | 8.62 | 1.052503 | 3.38 |
| 2 | 2 | 1.84 | 32 | 241.4 | 221.3 | 25.3 | 85 | 3.66 | 0.768148 | 98 | 185 | 121 | 44 | 3 | 4.58 | 1.526667 | 5.69 |
| 2 | 2 | 1.08 | 26 | 338 | 93.1 | 31.65 | 82 | 8.91 | 1.804 | 204 | 239 | 101 | 76 | 6.9 | 33.17 | 4.807246 | 5.05 |
| 2 | 2 | 1.42 | 32 | 281 | 103.4 | 28.2 | 90 | 5.78 | 1.284444 | 100 | 190 | 98 | 57 | 9.35 | 21.15 | 2.262032 | 2.22 |
| 2 | 2 | 1.3 | 27 | 149.6 | 310.5 | 32.02 | 113 | 10.9 | 3.041235 | 216 | 207 | 123 | 37 | 5.8 | 6.28 | 1.082759 | 4.33 |
| 2 | 2 | 2.31 | 26 | 619.8 | 219.4 | 27.93 | 88 | 2.55 | 0.554074 | 94 | 145 | 94 | 37 | 4.39 | 4.01 | 0.91344 | 3.94 |
| 2 | 2 | 2.05 | 35 | 223.8 | 194 | 26.5 | 92 | 2.67 | 0.606519 | 168 | 197 | 121 | 51 | 3.13 | 4.9 | 1.565495 | 4.13 |
| 2 | 2 | 2.41 | 28 | 752.2 | 316.6 | 29.41 | 107 | 2.144 | 0.565383 | 127 | 170 | 107 | 40 | 9.1 | 18.22 | 2.002198 | 3.19 |
| 2 | 2 | 3.76 | 23 | 738.4 | 245.4 | 28.09 | 90 | 3.56 | 0.791111 | 92 | 124 | 67 | 45 | 8.37 | 15.5 | 1.851852 | 3.16 |
| 2 | 2 | 3.87 | 29 | 627.4 | 133.2 | 25.43 | 85 | 2.05 | 0.430247 | 109 | 167 | 101 | 52 | 10.84 | 54.21 | 5.000923 | 1.39 |
| 2 | 2 | 2.89 | 31 | 682.1 | 281.9 | 28.99 | 86 | 2.44 | 0.518123 | 100 | 180 | 100 | 46 | 12.21 | 34.01 | 2.785422 | 2.28 |
| 2 | 2 | 2.85 | 22 | 491.4 | 210.4 | 25.41 | 105 | 5.62 | 1.457037 | 117 | 121 | 78 | 44 | 10.84 | 49.31 | 4.548893 | 4.01 |
| 2 | 2 | 1.86 | 34 | 214.9 | 220.3 | 27.44 | 105 | 4.77 | 1.236667 | 104 | 170 | 97 | 53 | 6.1 | 25.42 | 4.167213 | 2.71 |
| 2 | 2 | 1.3 | 38 | 488.2 | 271 | 29.88 | 97 | 6.45 | 1.544815 | 109 | 198 | 120 | 39 | 6.42 | 10.36 | 1.613707 | 4.33 |
| 2 | 2 | 1.85 | 30 | 331.6 | 124 | 25.66 | 84 | 4 | 0.82963 | 105 | 190 | 102 | 53 | 10.02 | 47.67 | 4.757485 | 2.36 |
| 2 | 2 | 3.16 | 25 | 874.4 | 331.5 | 26.4 | 83 | 2.59 | 0.53079 | 69 | 132 | 76 | 42 | 6.15 | 20.55 | 3.341463 | 2.19 |
| 2 | 2 | 1.96 | 33 | 327.1 | 92.8 | 27.44 | 103 | 4.85 | 1.233457 | 143 | 197 | 130 | 48 | 6.15 | 13.11 | 2.131707 | 3.07 |
| 2 | 2 | 1.3 | 35 | 288.9 | 92 | 29.05 | 103 | 8.14 | 2.070173 | 148 | 137 | 89 | 38 | 7.38 | 21.07 | 2.855014 | 3.1 |
| 2 | 2 | 2.14 | 31 | 237.8 | 124.9 | 26.54 | 114 | 10.37 | 2.918963 | 118 | 206 | 142 | 36 | 3.54 | 3.74 | 1.056497 | 2.31 |
| 2 | 2 | 1.46 | 29 | 331.5 | 304.9 | 28.75 | 102 | 6.41 | 1.61437 | 123 | 189 | 98 | 35 | 8.65 | 36.05 | 4.16763 | 2.45 |
| 2 | 2 | 3.2 | 21 | 331.4 | 238.5 | 25.33 | 108 | 6.22 | 1.658667 | 189 | 231 | 164 | 44 | 4.39 | 11.52 | 2.624146 | 4.68 |
| 2 | 2 | 2.8 | 21 | 374.8 | 184.5 | 25.1 | 75 | 3.3 | 0.611111 | 139 | 188 | 135 | 34 | 5.03 | 17.98 | 3.574553 | 4.23 |
| 2 | 2 | 2.07 | 27 | 389.5 | 89.5 | 26.52 | 82 | 3.95 | 0.799753 | 120 | 192 | 105 | 39 | 5.76 | 9.74 | 1.690972 | 3.24 |
